# High *E2F7* Expression Indicates Pancreatic Cancer Aggressiveness and Downregulation of *E2F7* Enhances Sensitivity to S-1

**DOI:** 10.1245/s10434-025-18912-3

**Published:** 2025-12-26

**Authors:** Keizo Fujita, Yukiyasu Okamura, Masamichi Hayashi, Ryo Ashida, Tomohisa Otsu, Akihiro Sakai, Shinji Mii, Motokazu Sugimoto, Naoto Yamamoto, Hirochika Toyama, Atsuyuki Maeda, Nobumasa Mizuno, Yukihiro Yokoyama, Junpei Yamaguchi, Nobuhiko Nakagawa, Keisuke Kurimoto, Haruyoshi Tanaka, Hideki Takami, Atsushi Enomoto, Katsuhiko Uesaka, Mitsuro Kanda

**Affiliations:** 1 Department of Gastroenterological Surgery, Nagoya University Graduate School of Medicine, Nagoya, Japan; 2 Division of Digestive Surgery, Department of Surgery, Nihon University School of Medicine, Tokyo, Japan; 3 Division of Hepato-Biliary-Pancreatic Surgery, Shizuoka Cancer Center, Shizuoka, Japan; 4 Department of Pathology, Nagoya University Graduate School of Medicine, Nagoya, Japan; 5 Department of Molecular Pathology, Graduate School of Biomedical and Health Sciences, Hiroshima University, Hiroshima, Japan; 6 Department of Hepatobiliary-Pancreatic Surgery, National Cancer Center Hospital East, Kashiwa, Japan; 7 Department of Gastrointestinal Surgery, Kanagawa Cancer Center, Yokohama, Japan; 8 Department of Surgery, Division of Hepato-Biliary-Pancreatic Surgery, Kobe University Graduate School of Medicine, Kobe, Japan; 9 Department of Surgery, Ogaki Municipal Hospital, Ogaki, Japan; 10 Department of Gastroenterology, Aichi Cancer Center Hospital, Nagoya, Japan; 11 Division of Surgical Oncology, Department of Surgery, Nagoya University Graduate School of Medicine, Nagoya, Japan

**Keywords:** Pancreatic cancer, *E2F7*, S-1, Gemcitabine

## Abstract

**Background:**

Pancreatic cancer (PC) remains a highly lethal disease with few reliable biomarkers to guide chemotherapy choices. New biomarkers for selecting anticancer drugs are needed to enhance the effectiveness of current multimodal treatment approaches. This study aimed to find a new biomarker by using clinical data and specimens collected for a Japanese randomized controlled trial (RCT).

**Methods:**

Gene expression array analysis was performed using PC tissues collected for the ancillary research of JASPAC01, a nationwide phase 3 RCT of adjuvant chemotherapy for patients with PC in Japan. A candidate gene was validated using tissue and blood samples from a second PC patient cohort undergoing radical surgery at the authors’ institution. Additionally, experiments were performed with cancer cell lines to investigate the functions of the candidate gene.

**Results:**

Expression of *E2F7* mRNA was the most influential prognostic factor of postoperative overall survival outcomes in the primary tissue-available cases in the JASPAC01 cohort (hazard ratio [HR], 1.386; 95% confidence interval [CI], 1.005–1.912; *p* = 0.045). High *E2F7* expression itself correlates with poor survival outcomes (*p* = 0.045 for OS). Moreover, the benefits of adjuvant S-1 treatment were reduced in high *E2F7* cases (*p* = 0.042 for OS; *p* = 0.007 for RFS). *In vitro* experiments demonstrated that *E2F7* inhibition suppressed cancer cell proliferation and minimized the 50% inhibitory concentration of S-1.

**Conclusions:**

Tissue mRNA expression levels of *E2F7* correlated with patient prognosis in PC. In the low *E2F7* mRNA expression group, patients who received S-1 as adjuvant chemotherapy had a better prognosis than those who received gemcitabine (GEM).

**Supplementary Information:**

The online version contains supplementary material available at 10.1245/s10434-025-18912-3.

Pancreatic cancer (PC) is a severe malignant tumor with an overall 5-year survival rate no higher than 5%.^1^ Because of the difficulty detecting the disease at an early stage, only 20% of patients receive a diagnosis of resectable PC. Even in these resectable cases, high recurrence rates occur, and almost 80% experience a recurrence.^2^ Therefore, patients who have undergone curative resection usually receive adjuvant chemotherapy to prevent early recurrence.

In Japan, the JASPAC01 study, a phase 3 randomized controlled trial (RCT), demonstrated the superiority of S-1 over gemcitabine (GEM) as adjuvant chemotherapy for 6 months in patients with PC. Based on the results of this study,^3^ S-1 is recommended by the Japan Pancreas Society as the first choice of adjuvant chemotherapy for PC.^4^ The recommended S-1 contains tegafur, a prodrug of 5-fluorouracil (5-FU) and gimeracil (CDHP), inhibits dihydropyrimidine dehydrogenase and is expected to enhance the cytotoxicity of 5-FU by prolonging high 5-FU concentrations in blood and tumor tissues.^5^

Because of the JASPAC01 study, the prognosis of patients with PC has improved significantly, but many patients are experiencing recurrence of PC. We need biomarkers that predict the effectiveness of GEM and S-1, and we planned this study as ancillary research of JASPAC01 to identify such biomarkers.

Among candidate genes, we focused on E2F transcription factor 7 (*E2F7*). Recently, E2F7 was identified as a member of the E2F family, a group of transcription factors involved in regulating the cell cycle, DNA replication, and cell senescence.^6^ As a family of transcription factors, E2F is involved in various cellular processes, including cell cycle, DNA damage response, and angiogenesis.

Transcription factors of the E2F family can be divided into typical E2Fs and atypical E2Fs. According to their functions, typical E2Fs can be divided into transcriptional activators (E2F1, E2F2, and E2F3a) and repressors (E2F3b, E2F4, E2F5, and E2F6).^6^ Atypical E2Fs include E2F7 and E2F8, which are potent repressors of E2F-dependent transcription.^7^

Several studies in solid cancers have demonstrated the involvement of E2F7 in chemoresistance. For instance, E2F7 induced doxorubicin resistance in head and neck squamous cell carcinoma^8^ and GEM resistance in lung adenocarcinoma.^9^ In PC, several reports indicate that E2F7 overexpression is associated with PC progression,^10–12^ whereas the effect of E2F7 expression levels on chemotherapy resistance remains unclear. This study aimed to elucidate the association between *E2F7* expression and the progression of PC or chemoresistance.

## Materials and Methods

This retrospective study conformed to the REporting recommendation for tumor MARKer prognostic studies (REMARK) guideline.^13^

### Candidate Genes

We first analyzed 22 genes as prognosticators (Table S1), which consisted of 14 gene candidates reported to be PC prognosis-related genes^14–27^ and 8 genes selected from the Gene Expression Omnibus (GSE28735) dataset analysis.

### GSE28735 Dataset Analysis

To select genes for analysis in the JASPAC01 study specimens, we investigated the public PC dataset (GSE28735). It provides microarray gene-expression profiles (using Affymetrix GeneChip Human Gene 1.0 ST array) of 45 matching pairs of pancreatic tumor and adjacent non-tumor tissues from 45 patients with pancreatic ductal adenocarcinoma. We selected candidate genes whose expression in tumor tissue was more than 1.35-fold higher than in normal tissue (*p* < 0.005, Welch’s *t* test; Table S2).

### Analysis of the JASPAC01 Samples

We collected unstained slides from formalin-fixed, paraffin-embedded (FFPE) surgical resection specimens from 24 institutions participating in the JASPAC01 study.^3^ The eligibility criteria for participants specified histologically proven invasive ductal carcinoma of the pancreas, excluding cystadenocarcinoma. A total of 377 patients who underwent macroscopic curative resection for pancreatic cancer were finally recruited between April 2007 and June 2010 (Fig. S1). The ethics committee of the Shizuoka Cancer Center institutional review board approved the protocol of this study (no. 27-22-2021-1-2).

We dissected the FFPE specimen sections (thickness, 10 µm) and cored out the cancerous tissues while referencing the hematoxylin–eosin-stained guiding slide. From these tissues, RNA was extracted and cDNA was synthesized. Then, polymerase chain reaction (PCR) for pre-amplification was performed using the PreAmp Master Mix Kit (Applied Biosystems, Waltham, MA, USA). We performed quantitative reverse transcription (qRT)-PCR by TaqMan Array Card (Applied Biosystems). The relative expression levels of target genes were calculated using the 2^−ΔΔCt^ method. As an internal control, glyceraldehyde-3-phosphate dehydrogenase (GAPDH) was used. We divided the patients into two groups based on the median expression level of each gene, then compared the prognosis of the two groups using a Cox proportional hazards model (Table 1).

**Table 1:** Correlation between the expression levels of 22 genes and OS

Gene^a^	HR (95% CI)	*p* Value^b^
***E2F7***	1.386 (1.005–1.912)	**0.045**
*TYMS*	1.371 (1.047–1.796)	**0.022**
*KRAS*	1.300 (0.972–1.739)	0.077
*VEGFA*	1.196 (0.915–1.564)	0.190
***CDKN3***	1.151 (0.879–1.509)	0.307
*SPARC*	1.146 (0.876–1.497)	0.320
***FOXM1***	1.122 (0.857–1.469)	0.403
***WDHD1***	1.116 (0.844–1.476)	0.442
***TUBB***	1.109 (0.848–1.451)	0.448
*HER2*	1.086 (0.831–1.420)	0.545
*CDKN2A*	1.063 (0.775–1.459)	0.704
*EGFR*	1.052 (0.804–1.377)	0.711
***DSG2***	1.029 (0.786–1.347)	0.836
*ELAVL1*	1.024 (0.782–1.340)	0.866
*UMPS*	0.967 (0.737–1.270)	0.811
***ADAM 19***	0.956 (0.730–1.253)	0.746
*RPM1*	0.919 (0.700–1.205)	0.540
*SMAD4*	0.899 (0.679–1.165)	0.394
*SLC29A1*	0.892 (0.683–1.166)	0.405
***CIT***	0.890 (0.679–1.165)	0.396
*TP53*	0.835 (0.638–1.092)	0.187
*DPYD*	0.804 (0.615–1.052)	0.112

HR, hazard ratio; CI, confidence interval

^a^GSE28735 analysis-derived eight genes are shown in bold style

^b^*p* Values below 0.05 are shown in bold style

### Analysis of PC Patients’ Specimens from Nagoya University Hospital

To validate the impact of E2F7 on S-1 administration, we analyzed serum samples and surgically resected specimens from patients with a diagnosis of resectable PC who received S-1-based neoadjuvant chemotherapy (two courses of GEM and S-1 therapy) and then underwent curative resection (R0) at Nagoya University Hospital between June 2020 and August 2024. The patients received adjuvant S-1 therapy, except when they were regarded as unable to tolerate chemotherapy or refused the treatment. All patients provided written informed consent to undergo surgery and research sample collection.

Since 2020, patients with resectable PC at Nagoya University Hospital (Nagoya, Japan) have been recommended to receive two courses of GEM and S-1 therapy as neoadjuvant chemotherapy (NAC-GS) and have received it if they agreed because NAC-GS has been commonly administered in Japan based on the Prep-02/JSAP-05 study^28,29^ and the Japan Pancreas Society guidelines.^30^

We collected clinical information from medical records. Regarding histopathologic assessment of the NAC-GS treatment effect, pathologists assessed the percentage of remaining viable cancer cells according to the Japanese classification of pancreatic carcinoma.^31^

### Serum E2F7 Protein Concentration

Blood samples were collected before the patients received NAC-GS. The samples were centrifuged at 3000 rpm (1710 g) for 5 min. The sera were transferred to fresh tubes and stored at −80 °C until analysis. Concentrations of E2F7 protein in sera were determined using a human E2F7 ELISA kit (MyBiosource, Inc., San Diego, CA, USA). Samples were tested in duplicate according to the manufacturer’s protocol, and the mean values were used for analysis.

### E2F7 mRNA Expression in PC Tissues

Pancreatic cancer tissues cored from surgically resected specimens were immediately frozen in liquid nitrogen and stored at −80 °C. Total RNA was isolated from these samples using the RNeasy Mini kit (Qiagen, Hilden, Germany), and the quantity and quality of the total RNA were verified with a Nanodrop spectrophotometer (Thermo Fisher Scientific, Waltham, MA, USA). Complementary DNA (cDNA) was synthesized using qScript cDNA SuperMix (Quanta Biosciences, Beverly, MA, USA) with 0.3 µg total RNA. We performed qRT-PCR using SYBR Premix Ex Taq II (Takara Bio Inc., Shiga, Japan) on an ABI Step One Plus Real-Time PCR System (Applied Biosystems). The thermocycling conditions were as follows: one cycle of 95 °C for 10 s, followed by 40 cycles of 95 °C for 5 s and 60°C for 30 s. Reactions were run in triplicate. The relative expression of genes was calculated using the 2^−ΔΔCt^ method with normalization to *ACTB*. The primer sequences were as follows: *E2F7* forward, 5′-AAAAGACCTGATCAGCCCCA-3′ reverse, 5′-TTCAGGTTAGCTGTGGGTGT-3′; and *ACTB* forward, 5′-CACCATTGGCAATGAGCGGTTC-3′ reverse, 5′-AGGTCTTTGCGGATGTCCACGT-30′.

### Cell Culture and Transfection

The study obtained PA-TU-8902, MIA PaCa-2, and SW1990 cell lines from the American Type Culture Collection (Manassas, VA, USA). Cancer cell lines were cultured in RPMI 1640 medium supplemented with penicillin, streptomycin, and 10% fetal bovine serum (Thermo Fisher Scientific) at 37 °C in a 5% CO_2_ incubator.

For RNAi experiments, each cell line was transfected with Silencer Select siRNA *E2F7* (Thermo Fisher Scientific) or Silencer Select Negative Control #1 siRNA using Lipofectamine RNAiMAX (Thermo Fisher Scientific), following the manufacturer’s protocol. Transfected cells were collected 48 h after transfection and subjected to downstream assays. We isolated RNA using the RNeasy Mini Kit (Qiagen, Hilden, Germany).

### Cell Proliferation Assay

Transfected cells (2 × 10^3^ cells/well) were seeded into a 96-well plate and cultured overnight. Then, cell proliferation was measured at different time points (0, 24, 48, and 72 h) using a Cell Counting Kit-8 (CCK-8) (Dojindo, Kumamoto, Japan). Reagent (10 µL/well) was added to each well, and the cells were incubated in the dark at 37 °C for 2 h. After incubation, the absorbance value at 450 nm was measured using spectrophotometric readings.

The study also used CCK-8 to assay the sensitivity of PC cells to anticancer drugs. We purchased GEM, 5-FU, and CDHP from Tokyo Chemical Industry Co., Ltd. (Tokyo, Japan). The 5-FU/CDHP mixture was used at a molar ratio of 1:1, which mimics human plasma pharmacokinetics after oral administration of S-1.^32^ After overnight incubation of transfected cells in a 96-well plate, 10-µl volumes of stepwise drug concentrations (0, 0.4, 2, 10, 50, 250 µM S-1 for PA-TU-8902 and MIA PaCa-2, determined based on past reports^33–35^) were added. After treatment with each drug for 72 h, CCK-8 assays were performed.

### Statistical Analysis

The chi-square test was used for comparisons between the categorical variables. The differences between groups were analyzed using Student’s *t* test. The survival rate was calculated using the Kaplan–Meier method and compared using the log-rank test. Uni- and multivariate Cox proportional hazard regression analyses were performed to evaluate independent prognostic factors associated with survival. All statistical analyses were performed using JMP Pro 16 software (SAS, Cary, NC, USA), and *p* values lower than 0.05 was considered statistically significant.

## Results

### Candidate Gene Selection

GSE28735 dataset analyses extracted eight genes (*ADAM19*, *CDKN3*, *CIT*, *DSG2*, *E2F7*, *FOXM1*, *TUBB3*, and *WDHD1*) that had higher mRNA expression levels in PC tissues than in adjacent non-tumor tissues and also correlated with the postoperative prognosis (Table 2). We added 14 well-known genes (*CDKN2A*, *DPYD*, *EGFR*, *ELAVL1*, *ERBB2*, *KRAS*, *RPM1*, *SLC29A1*, *SMAD4*, *SPARC*, *TP53*, *TYMS*, *UMPS*, and *VEGFA*) to the analysis by referencing past reports. A total of 22 genes were chosen as oncogenic candidates, and further analysis was performed using the JASPAC01 samples (Table S1).

**Table 2:** Correlation between E2F7 mRNA expression level and clinicopathologic features

Clinicopathologic features	*E2F7* mRNA High-expression group (*n* = 111)	*E2F7* mRNA Low-expression group (*n* = 110)	*p* value
Sex			0.640
Male	60	56	
Female	51	54	
Age (years)			0.770
Mean ± SD	66.1 ± 8.8	65.7 ± 8.7	
Performance status			0.069
PS0	68	80	
PS1	43	30	
Preoperative CEA (ng/ml)			0.323
Mean ± SD	2.5 ± 2.1	2.8 ± 2.9	
Preoperative CA19-9 (U/ml)			0.697
Mean ± SD	88.4 ± 413.5	126.5 ± 915.1	
Primary tumor status			0.306
* T* < 3	12	17	
* T* ≥ 3	99	93	
Regional lymph node status			0.111
N0	34	45	
N1	77	65	
Residual tumor status			0.182
R0	92	98	
R1	19	12	
Adjuvant chemotherapy regimen			0.051
GEM	63	48	
S-1	48	62	

*SD* standard deviation

### Candidate Gene Expression Levels and Prognosis in the JASPAC01 Study Cohort

We measured candidate gene mRNA expression levels in resected cancer tissues from 221 available patients (58.6%) of the JASPAC01 study (Fig. S1). This sample cohort consisted of almost the same number of GEM-administered cases and S-1-administered cases with similar clinical characteristics (Table S3). Among the candidates, only *E2F7* (*p* = 0.045) and *TYMS* (*p* = 0.022) showed statistically significant correlations with OS (Table 1). We then focused on *E2F7* in the current study because its hazard ratio (HR) was the highest (HR, 1.386; 95% confidence interval [CI], 1.005–1.912), and TYMS has already been reported to be a predictor of 5-FU sensitivity in PC.^16^

### E2F7 Expression and Postoperative Survival Outcomes

**Fig. 1:**
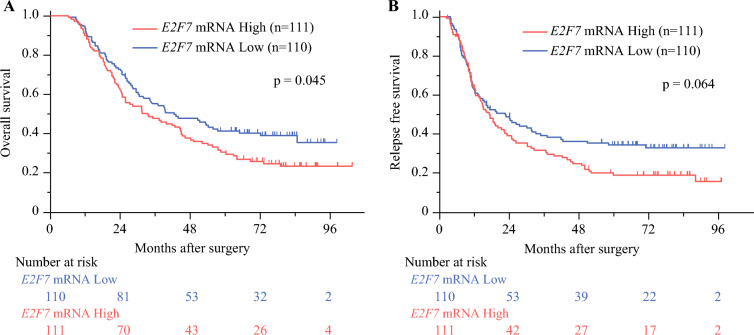
**A** Kaplan–Meier survival curves for overall survival after stratification by *E2F7* expression. **B** Kaplan–Meier survival curves for relapse-free survival after stratification by *E2F7* expression

Among the entire cohort, high *E2F7* mRNA expression cases demonstrated significantly worse overall survival (OS) outcomes (*p* = 0.045) and marginally worse recurrence-free survival (RFS) outcomes (*p* = 0.064) (Fig. 1). Regarding cohort background, no significantly different clinical factors were detected between the high and low *E2F7* mRNA expression groups (Table 2).

**Fig. 2:**
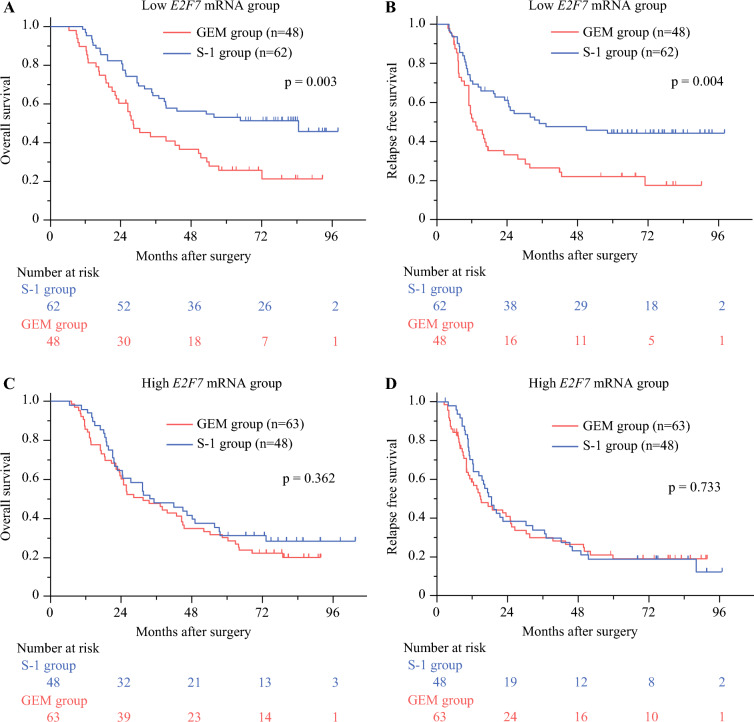
**A** Kaplan–Meier survival curves for overall survival in the low *E2F7* mRNA expression group after stratification by treatment. **B** Kaplan–Meier survival curves for relapse free survival in the low *E2F7* mRNA expression group after stratification by treatment. **C** Kaplan–Meier survival curves for overall survival in the high *E2F7* mRNA expression group after stratification by treatment. **D** Kaplan–Meier survival curves for relapse free survival in the high *E2F7* mRNA expression group after stratification by treatment

We divided the entire cohort with the median value of the *E2F7* mRNA expression level and analyzed each of the low and high *E2F7* mRNA expression groups (Fig. 2). Among the low *E2F7* mRNA expression group, the prognosis of patients receiving S-1 as adjuvant chemotherapy was better than that of those receiving GEM (*p* = 0.003 for OS; *p* = 0.004 for RFS), as in the JASPAC01 cohort. However, no statistically significant difference was observed in the high *E2F7* mRNA expression group (*p* = 0.362 for OS; *p* = 0.733 for RFS). This may imply that the superiority of the S-1 treatment effect in the adjuvant setting was diminished in the high *E2F7* mRNA expression group.

**Fig. 3:**
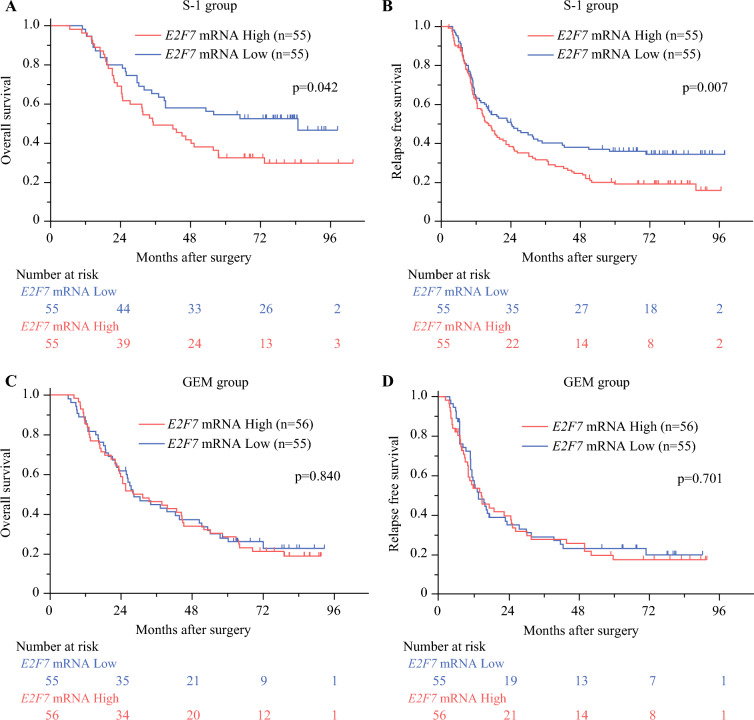
**A** Kaplan–Meier survival curves for overall survival in the S-1 arm after stratification by *E2F7* expression. **B** Kaplan–Meier survival curves for relapse-free survival in the S-1 arm after stratification by *E2F7* expression. **C** Kaplan–Meier survival curves for overall survival in the gemcitabine (GEM) arm after stratification by *E2F7* expression. **D** Kaplan–Meier survival curves for relapse-free survival in the GEM arm after stratification by *E2F7* expression

We also performed a subgroup analysis among patients who received each chemotherapy treatment (Fig. 3). We observed that high *E2F7* mRNA expression cases showed worse survival outcomes in S-1-administered cases (*p* = 0.042 for OS; *p* = 0.007 for RFS), whereas no difference was identified in GEM-administered cases (*p* = 0.840 for OS; *p* = 0.701 for RFS). These results suggest that high mRNA status may attenuate the effects of S-1 treatment. In the S-1 arm, the multivariate analysis showed that high *E2F7* mRNA expression was a significant predictor of RFS (HR, 1.854; 95% CI, 1.091–3.151; Table 3).

**Table 3:** Uni- and multivariable Cox proportional hazard regression analysis of relapse-free survival in the S-1 arm of the study

Variables	Univariate analysis		Multivariable analysis	
	HR (95% CI)	*p* value	HR (95% CI)	*p* value
Sex (male vs female)	0.975 (0.615–1.547)	0.914		
Age (≥ 65 vs < 65)	1.130 (0.707–1.806)	0.611		
Performance status (PS1 vs PS0)	1.186 (0.714–1.970)	0.511		
Chemotherapy protocol (incomplete vs complete)	1.426 (0.865–2.353)	0.164	1.370 (0.810–2.319)	0.241
Preoperative CA19-9 (high vs low)^a^	1.701 (1.051–2.753)	0.031	1.602 (0.976–2.629)	0.062
Primary tumor status (*T* ≥ 3 vs *T* < 3)	1.643 (0.753–3.587)	0.212		
Regional lymph node status (N1 vs N0)	1.963 (1.150–3.350)	0.013	1.560 (0.873–2.788)	0.133
Residual tumor status (R1 vs R0)	1.734 (0.927–3.245)	0.085	1.570 (0.827–2.982)	0.168
*E2F7* mRNA expression (high vs low)^a^	1.889 (1.178–3.028)	0.008	1.854 (1.091–3.151)	**0.023**

Bold indicates *p* < 0.05 factors

*HR* hazard ratio, *CI* confidence interval

^a^Divided by the median value in the S-1 arm

### Neoadjuvant Treatment Effect and Prognosis Analysis Using PC Cases at Nagoya University Hospital

To further elucidate the impact of E2F7 on the S-1 treatment effect, we examined *E2F7* mRNA expression levels in 40 surgical specimens from patients undergoing NAC-GS. We also examined E2F7 concentration in 16 pretreatment serum samples among the 40 patients (Table S4). Regarding cohort background, no significant differences in clinical factors were observed between the high and low *E2F7* mRNA expression groups (Table S5).

**Fig. 4:**
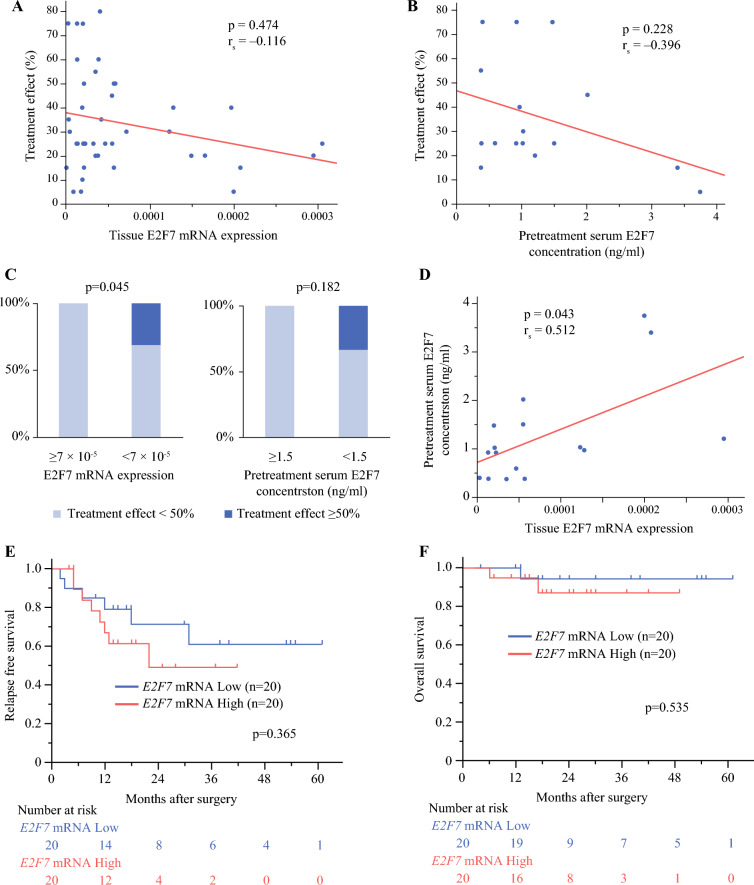
**A** Spearman correlation between NAC-GS treatment effect and surgically resected tissue *E2F7* mRNA expression level. The histologic NAC-GS treatment effect was plotted on the y-axis, and the tissue E2F7 mRNA expression levels were plotted on the x-axis. **B** Spearman correlation between NAC-GS treatment effect and pretreatment serum E2F7 concentration. The histologic NAC-GS treatment effect was plotted on the y-axis, and the pretreatment serum E2F7 concentrations were plotted on the x-axis. **C** Proportion of patients with treatment effects more than 50% or less than 50% according to *E2F7* mRNA expression level and pretreatment serum E2F7 concentration divided by the third quartile. **D** Spearman correlation between surgically resected tissue *E2F7* mRNA expression level and pretreatment serum E2F7 concentration. **E** Kaplan–Meier survival curves for relapse-free survival after stratification by E2F7 expression. **F** Kaplan–Meier survival curves for overall survival after stratification by E2F7 expression. NAC-GS, gemcitabine and S-1 therapy as neoadjuvant chemotherapy

There was no statistically significant correlation between the tissue E2F7 mRNA expression levels and the treatment effect of NAC-GS (Fig. 4A). The same was true for the pretreatment E2F7 concentration in serum (Fig. 4B). However, in both analyses, the treatment effects of more than 50% were not observed among the patients with E2F7 levels above the third quartile (Fig. 4C). Pretreatment serum E2F7 concentration was correlated with surgical specimen *E2F7* mRNA expression level (Fig. 4D).

We divided the 40 patients into two groups based on the median E2F7 mRNA expression level and analyzed survival. We observed no statistically significant correlation between the tissue *E2F7* mRNA expression levels and the prognosis (Fig. 4E and F).

### E2F7 Knockdown and Overexpression in PC Cell Lines

**Fig. 5:**
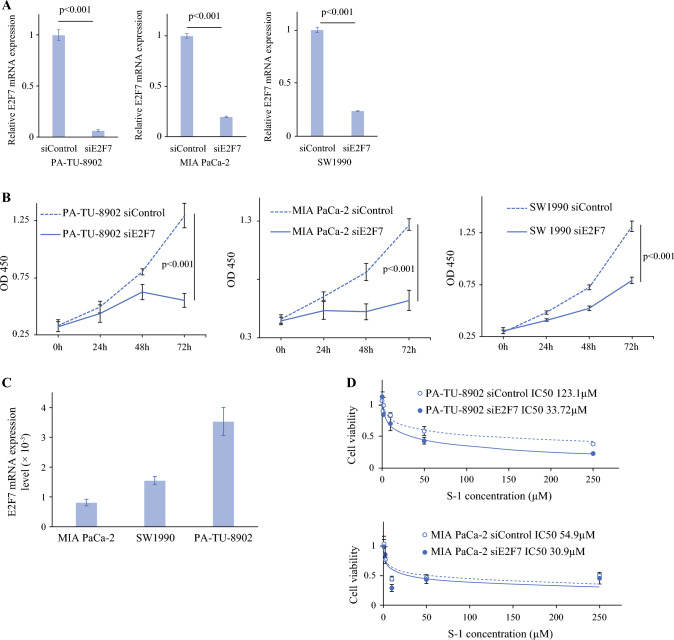
Effects of E2F7 on proliferation and chemotherapy sensitivity of PC cells. **A** Relative *E2F7* mRNA expression levels normalized to *ACTB* after *E2F7* siRNA transfection. **B** Proliferation ability of PC cells after *E2F7* siRNA transfection. **C** Relative *E2F7* mRNA expression level of each untransfected PC cell line. **D** IC50 values of PC cells treated with S-1 after *E2F7* siRNA transfection

Next, we attempted to replicate the correlation between E2F7 expressions and the effects of S-1 treatment in an *in vitro* experiment. We used three PC cell lines and induced knockdown transfection (Fig. 5A). The *E2F7* knockdown led to apparent PC cell proliferation inhibition (Fig. 5 B).

To evaluate the anticancer ability of the drug, we assessed IC50 values in PA-TU-8902 and MIA PaCa-2 cells with *E2F7* knockdown. The *E2F7*-silenced PA-TU-8902 cells showed increased sensitivity to S-1 compared with MIA PaCa-2 cells. These results demonstrated that E2F7 inhibition enhanced S-1 sensitivity, particularly in cells with initially high *E2F7* mRNA expression (Fig. 5C and D).

## Discussion

In PC, there are currently no widely used biomarkers for predicting drug efficacy, such as the KRAS mutation in colorectal cancer^36^ and the estrogen receptor in breast cancer.^37^ In the current study, we analyzed clinical data and preserved specimens from the JASPAC01 study to identify PC biomarkers that predict prognosis and chemotherapy effectiveness. Among 22 candidate genes, *E2F7* and *TYMS* showed a correlation with prognosis. Thymidylate synthase (TYMS) is a target of 5-FU, and an earlier study reported that its expression level predicts response to 5-FU-based adjuvant chemotherapy in PC.^16^ As in this previous report, patients with high *TYMS* mRNA expression levels had a worse prognosis than those with low expression, and the difference was observed only among patients who received S-1 (the prodrug of 5-FU) as adjuvant chemotherapy (data not shown). However, to the best of our knowledge, there are no previous reports that *E2F7* expression level predicts the effectiveness of PC chemotherapy.

Recent studies have reported that E2F7 is attracting attention and promotes tumorigenesis in various cancer types.^6,10,11^ In other words, downregulation of *E2F7* led to a significant suppression of cancer cell proliferation. Our results and previous reports have shown that PC,^11^ breast cancer,^38^ and lung cancer^9^ cells exhibit suppression of proliferation after the knockdown of *E2F7*. However, downregulation of E2F7 in gastric^39^ and skin cancer^40^ cells has been shown to accelerate proliferation. Thus, the oncogenic function of E2F7 appears to depend on the type of cancer cell.

The results of the current study also suggest that E2F7 expression levels influence the efficacy of chemotherapy, particularly S-1. We also confirmed that *E2F7* knockdown in PC cell lines increased sensitivity to S-1. The *E2F7* mRNA expression level in surgically resected specimens may be a helpful biomarker for selecting adjuvant chemotherapy drugs. Patients with high *E2F7* mRNA expressions may derive only marginal benefits from S-1.

Currently in Japan, patients with resectable PC are commonly recommended to receive neoadjuvant and adjuvant S-1-based chemotherapy, based on the Prep-02/JSAP-05 and JASPAC 01 studies.^3,29^ Regarding adjuvant S-1 therapy, patients with high *E2F7* mRNA expressions would need another RCT to determine the effectiveness of chemotherapies other than S-1. On the other hand, regarding NAC-GS, the correlation between the effectiveness and E2F7 expression levels remains unclear in the current study. However, this may be due to a small sample size. To use S-1, one of the key drugs in PC treatment, requires markers predictive of its anticancer effect, such as E2F7 expression. If a specific predictive marker were established, adequate S-1-based regimen selection could be more accurately determined, thereby prolonging disease-free survival.

Pretreatment serum E2F7 concentration was correlated with the *E2F7* mRNA expression level of resected specimens. Although E2F7 is a transcription factor and typically localizes to the perinuclear region, we detected it in the serum samples. Resected tissue samples are not available before neoadjuvant chemotherapy, and invasive examinations, such as endoscopic ultrasound-guided fine-needle biopsy, are required to evaluate E2F7 mRNA expression. Because blood sampling is less invasive than tissue sampling, serum E2F7 levels could serve as a biomarker to predict chemotherapy effects.

Our study had several limitations. Although we also conducted immunostaining of preserved FFPE specimens from the JASPAC01 study, evaluation was difficult due to specimen damage or inconsistent staining, likely caused by differences in specimen processing and storage methods at each institution (Fig. S2A and B). This is why we attempted to examine serum samples in our cohort. As another limitation, this study was retrospective and had a smaller sample size than the JASPAC01 study.

In conclusion, tissue mRNA expression levels of *E2F7* correlated with the prognosis of patients with PC. Notably, in the low *E2F7* mRNA expression group, patients who received S-1 as adjuvant chemotherapy had a better prognosis than those who received GEM.

## Supplementary Information

Below is the link to the electronic supplementary material.

Supplementary file1 (JPEG 118 KB)

Supplementary file2 (DOCX 266 KB)

Supplementary file3 (DOCX 33 KB)
