# Supplementary figures and images for: High E2F7 Expression Indicates Pancreatic Cancer Aggressiveness and Downregulation of E2F7 Enhances Sensitivity to S-1
Source: Ann Surg Oncol. 2025 Dec 26;33(6):5429–40. doi: 10.1245/s10434-025-18912-3 (PMC13179229; doi:10.1245/s10434-025-18912-3)

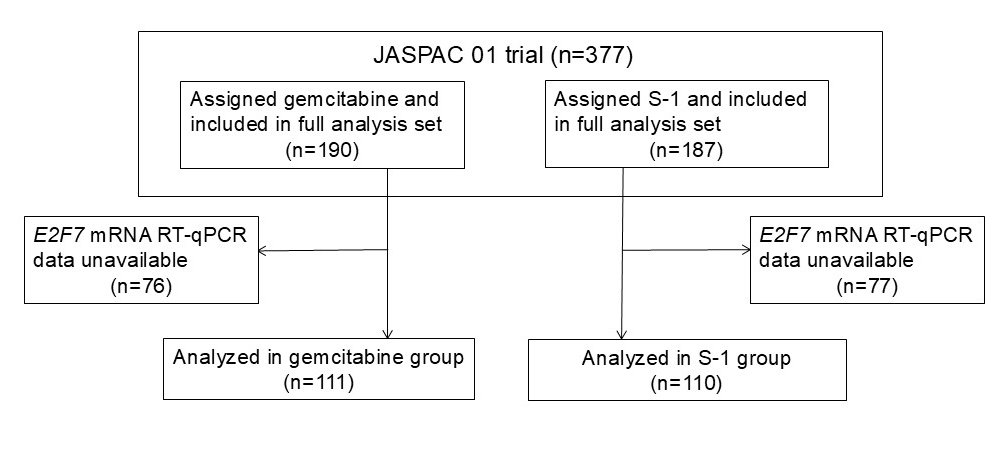

Supplement: Supplementary file 1 — Supplementary file1 (JPEG 118 KB) [file 10434_2025_18912_MOESM1_ESM.jpg]

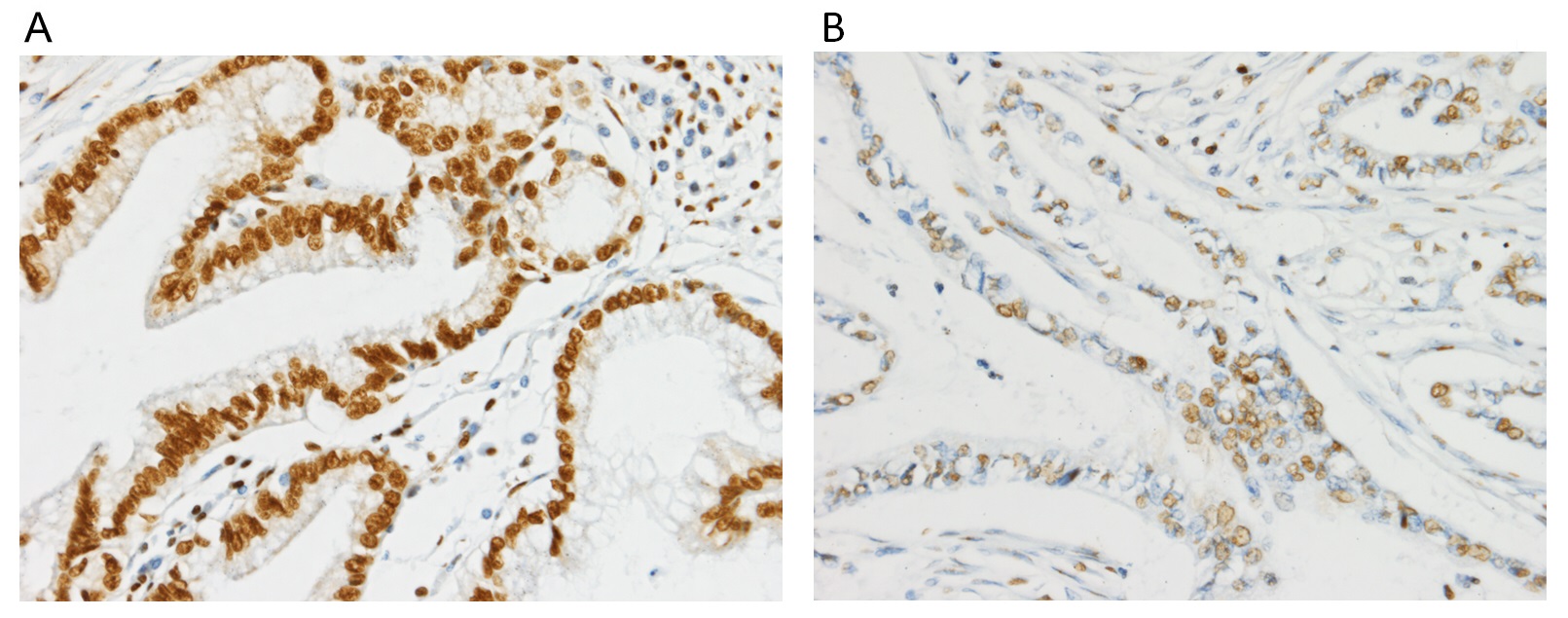

Supplement: Supplementary file 2 — Supplementary file2 (DOCX 266 KB) [file 10434_2025_18912_MOESM2_ESM.jpg]
