# Supplementary material for: High E2F7 Expression Indicates Pancreatic Cancer Aggressiveness and Downregulation of E2F7 Enhances Sensitivity to S-1
Source: Ann Surg Oncol. 2025 Dec 26;33(6):5429–40. doi: 10.1245/s10434-025-18912-3 (PMC13179229; doi:10.1245/s10434-025-18912-3)
Supplement: Supplementary file 3 — Supplementary file3 (DOCX 33 KB) [file 10434_2025_18912_MOESM3_ESM.docx]

**Supplementary Table 1**

List of analyzed 22 genes.

| Gene symbol | Description |
| --- | --- |
| ***ADAM19***  *CDKN2A / p16*  ***CDKN3***  ***CIT***  *DPYD*  ***DSG2***  ***E2F7***  *EGFR*  ***FOXM1***  *HER2*  *HuR*  *KRAS*  *RPM1*  *SMAD4 / DPD4*  *SLC29A1*  *SPARC*  *TYMS*  *TP53*  ***TUBB***  *UMPS*  *VEGFA*  ***WDHD1*** | ADAM metallopeptidase domain 19  Cyclin-dependent kinase inhibitor 2A  Cyclin-dependent kinase 3  Citron rho-interacting serine/threonine kinase  Dihydropyrimidine dehydrogenase  Desmoglein 2  E2F transcription factor 7  Epidermal growth factor receptor  Forkhead box M1  Human epidermal growth factor receptor 2  Intratumoral human antigen R  Kirsten rat sarcoma viral oncogene homolog  Ribonucleotide reductase M1  SMAD family member 4  Human equilibrative nucleoside transporter 1  Secreted protein, acidic, cysteine-rich  Thymidylate synthase  Tumor protein p53  Tubulin, beta class I  Orotate phosphoribosyl transferase  Vascular endothelial growth factor  WD repeat and HMG-box DNA binding protein 1 |

GSE28735 dataset analysis derived eight genes were shown in bold style.

**Supplementary Table 2**

Analysis of GSE28735 dataset.

| **Gene** | **Fold change (Tumor/Normal)** | **HR (95% CI)** | **Log-rank**  **p-value** |
| --- | --- | --- | --- |
| *ADAM 19*  *CDKN3*  *CIT*  *DSG2*  *E2F7*  *FOXM1*  *TUBB*  *WDHD1* | 1.80  1.40  1.42  2.19  1.53  1.59  1.47  1.56 | 3.36 (1.43-7.89)  3.78 (1.51-9.44)  4.75 (1.82-12.39)  3.32 (1.44-7.61)  4.23 (1.63-10.97)  3.87 (1.49-10.03)  3.06 (1.35-6.92)  3.68 (1.42-9.57) | **0.0031**  **0.0022**  **0.0005**  **0.0028**  **0.0012**  **0.0026**  **0.0046**  **0.0039** |

**Supplementary Table 3**

Characteristics of 221 patients whose *E2F7* mRNA expressions were measurable.

|  | **Total**  **221 cases** | **Gemcitabine group (n=111)** | **S-1 group (n=110)** |
| --- | --- | --- | --- |
| Sex  Male  Female  Age (years)  > 65  < 65  Performance status  0  1  Preoperative CEA (ng/ml)  Mean ± SD  Preoperative CA19-9 (U/ml)  Mean ± SD  Operative procedure  Pancreaticoduodenectomy  Distal pancreatectomy  Total pancreatectomy  Combined portal vein resection  Number of dissected lymph nodes (median, IQR)  Number of dissected lymph nodes (mean, SD)  Residual tumor status  R0  R1  Primary tumor status*  T1  T2  T3  T4  Regional lymph node status*  N0  N1 | 116 (52%)  105 (48%)  135 (61%)  86 (39%)  148 (67%)  73 (33%)  2.7 ± 2.5  107.0 ± 702.9  148 (67%)  72 (33%)  1  65 (29%)  24 (16-33.5)  26.5 (14.2)  190 (86%)  31 (14%)  15 (7%)  14 (6%)  191 (86%)  1  79 (36%)  142 (64%) | 55 (50%)  56 (50%)  71 (64%)  40 (36%)  68 (61%)  43 (39%)  2.5 ± 2.1  178.4 ± 991.2  80 (72%)  30 (27%)  1 (1%)  32 (29%)  27 (18-33)  27.3 (13.9)  95 (86%)  16 (14%)  6 (5%)  9 (8%)  96 (87%)  0  44 (40%)  67 (60%) | 61 (55%)  49 (45%)  64 (58%)  46 (42%)  80 (73%)  30 (27%)  2.8 ± 2.9  36.3 ± 70.8  68 (62%)  42 (38%)  0  33 (30%)  23 (15-34)  25.7 (14.6)  95 (86%)  15 (14%)  9 (8%)  5 (5%)  95 (86%)  1 (1%)  35 (32%)  75 (68%) |

*Primary tumor status and lymph node status were described according to the TNM Classification of malignant tumors, 6th edition

**Supplementary Table 4**

Characteristics of 40 patients who received NAC-GS and curative resection.

| **Characteristics** | **All patients (n=40)** | **Patients with pretreatment serum *E2F7* measured (n=16)** |
| --- | --- | --- |
| Sex  Male  Female  Age (years)  Mean ± SD  Pretreatment CEA (ng/ml)  Mean ± SD  Pretreatment CA19-9 (U/ml)  Mean ± SD  Operative procedure  Pancreaticoduodenectomy  Distal pancreatectomy  Total pancreatectomy  Other  Primary tumor status*  T1  T2  T3  T4  Regional lymph node status*  N0  N1a  N1b | 27 (68%)  13 (33%)  68.4 ± 9.1  3.5 ± 2.3  155.4 ± 227.9  17 (43%)  19 (48%)  3 (8%)  1 (3%)  5 (13%)  0  35 (88%)  0  24 (60%)  12 (30%)  4 (10%) | 10 (63%)  6 (38%)  70.9 ± 9.0  4.2 ± 2.8  246.6 ± 327.0  5 (31%)  9 (56%)  2 (13%)  0  2 (13%)  0  14 (88%)  0  12 (75%)  3 (19%)  1 (6%) |

*Primary tumor status and lymph node status were described according to the Japanese classification of pancreatic carcinoma by the Japan Pancreas Society: Eighth edition

**Supplementary table 5**

Correlation between E2F7 mRNA expression level and clinicopathological features.

| **Characteristics** | ***E2F7* mRNA**  **high expression group**  **(n=20)** | ***E2F7* mRNA**  **low expression group**  **(n=20)** | **p-value** |
| --- | --- | --- | --- |
| Sex  Male  Female  Age  Mean ± SD (years)  Preoperative CEA  Mean ± SD (ng/ml)  Preoperative CA19-9  Mean ± SD (U/ml)  Primary tumor status  T < 3  T > 3  Regional lymph node status  N0  N1  Adjuvant chemotherapy regimen  Complete  Incomplete or None | 13  7  68.6 ± 8.4  3.8 ± 2.8  151.3 ± 168.7  3  17  11  9  14  6 | 14  6  68.2 ± 10.4  3.1 ± 1.6  159.8 ± 282.1  2  18  13  7  13  7 | 0.736  0.907  0.404  0.909  0.633  0.519  0.736 |
